# pH-Responsive Nanoemulsions Based on a Dynamic Covalent Surfactant

**DOI:** 10.3390/nano11061390

**Published:** 2021-05-25

**Authors:** Gaihuan Ren, Bo Li, Lulu Ren, Dongxu Lu, Pan Zhang, Lulu Tian, Wenwen Di, Weili Shao, Jianxin He, Dejun Sun

**Affiliations:** 1 Textile and Garment Industry of Research Institute, Zhongyuan University of Technology, Zhengzhou 450007, China; ghren@zut.edu.cn (G.R.); 2020110366@zut.edu.cn (B.L.); tian543669@163.com (D.L.); 2019010180@zut.edu.cn (P.Z.); 2019110205@zut.edu.cn (L.T.); 2 Key Laboratory of Colloid and Interface Chemistry, Ministry of Education, Shandong University, Jinan 250100, China; 201912144@mail.sdu.edu.cn (L.R.); 201832281@mail.sdu.edu.cn (W.D.)

**Keywords:** pH-responsive, nanoemulsions, dynamic covalent bond

## Abstract

Developing solid-free nanoemulsions with pH responsiveness is desirable in enhanced oil recovery (EOR) applications. Here, we report the synthesis of an interfacial activity controllable surfactant (T−DBA) through dynamic imine bonding between taurine (T) and p-decyloxybenzaldehyde (DBA). Instead of macroemulsions, nanoemulsions can be prepared by using T−DBA as an emulsifier. The dynamic imine bond of T−DBA enables switching between the active and inactive states in response to pH. This switching of interfacial activity was used to gate the stability of nanoemulsions, thus enabling us to turn the nanoemulsions off and on. Using such dynamic imine bonds to govern nanoemulsion stability could enable intelligent control of many processes such as heavy oil recovery and interfacial reactions.

## 1. Introduction

In the petroleum industry, forming stable emulsions with small droplet sizes (nanoemulsions) is important so that the oil can pass through rock pores without becoming trapped during enhanced oil recovery (EOR). Successful demulsification of stable nanoemulsions is crucial for the effective recovery of the emulsified crude oil [1,2,3]. Thus, nanoemulsions with controllable stability are desired in EOR applications.

Responsive emulsifiers have emerged as a class of smart surfactants that permit regulating the emulsion stability under external stimuli [4,5,6,7,8]. The external stimuli include pH [9,10,11,12,13,14,15], CO_2_ [6,16], temperature [17], magnetism [8], light [18,19], and redox [7,20]. Among these external stimulation types, pH has attracted particular interest because it has the advantage of being simple to regulate [11,21]. The widely studied pH-responsive emulsifiers for EOR applications are particle-based [5,22,23]. However, solid particles in the flooding fluid can cause problems, such as permanent clogging of the rock pores, particularly when these pores are small or if the particles in the utilized Pickering emulsion tend to flocculate or aggregate [5,24]. Toxicity, environmental impact, and sustainability are also major concerns when choosing particle-based materials for EOR applications [1,5]. Thus, developing solid-free emulsifiers with pH responsiveness is more desirable for EOR applications.

Recently, several types of solid-free emulsifiers with pH responsiveness have been investigated [25,26]. Surfactants containing responsive groups such as amidine groups, amine groups, or carboxyl groups comprise a large category of pH-responsive emulsifiers. When the pH changes, the protonation or deprotonation of the responsive group in the molecule results in the surfactant switching between the active and inactive states, contributing to the pH-responsive nature of the emulsions. However, these surfactants usually require a complicated synthesis process [12,27]. Hence, there is a clear need to develop simple and eco-friendly pH-responsive surfactants.

The dynamic imine bond provides a new method for constructing surfactants with pH responsiveness and has already been widely used to fabricate pH-responsive surfactants due to its synthetic simplicity and pH-responsive nature [28,29,30]. Diverse forms of covalent surfactants based on the dynamic imine bond were reported by van Esch and coworkers. These surfactants were stable under basic conditions and dissociated under acidic conditions [31]. In our previous work, a pH-responsive dynamic covalent polymer emulsifier and dynamic covalent Pickering emulsifier were prepared for developing pH-responsive macroemulsions and Pickering emulsions, respectively [11,12]. The above emulsions contain micron-scale droplets [11,12]. In the petroleum industry, the oil must pass through rock pores without becoming trapped. Therefore, developing solid-free pH-responsive nanoscale emulsions is more desirable than micron-scale emulsions [32,33,34,35,36].

In this study, we demonstrate the fabrication of a dynamic covalent small-molecule surfactant (taurine–p-decyloxybenzaldehyde, T−DBA) for solid-free pH-responsive nanoemulsion preparation. The dynamic nature of the imine bond in T−DBA allows the manipulation of interfacial activity through pH changes. At pH = 12, when the interfacial tension (IFT) between liquid paraffin and water was decreased to 10 mN/m, a nanoemulsion was obtained with T−DBA as an emulsifier. As the pH decreased from 10 to 3, the IFT increased to approximately 34 mN/m, and the nanoemulsion was demulsified within 30 min. Such pH-responsive nanoemulsions are highly desirable in heavy oil recovery applications. This work provides theoretical and technical support for designing pH-responsive emulsifiers based on dynamic covalent bonds and their application in EOR.

## 2. Experimental Section

### 2.1. Chemicals and Materials

Taurine (T, 99% purity) was obtained from Shanghai Macklin Biochemical Co., Ltd., Shanghai, China. p-Decyloxybenzaldehyde (DBA, 97% purity) was provided by J&K Scientific Ltd., Beijing, China. Liquid paraffin was received from ExxonMobil Research and Engineering Company, (Irving, TX, USA). Potassium hydroxide (AR), toluene (AR, ≥99.5%), dichloromethane (AR, ≥99.5%) and hydrogen chloride (36.5 wt%) were purchased from Sinopharm Chemical Reagent Co. Ltd., Shanghai, China. *n*-Tetradecane (99% purity) was obtained from Aladdin Reagents (Shanghai, China). Crude oil was supplied by China Petroleum & Chemical Corporation (Beijing, China). All of the chemicals were used as received without further purification.

### 2.2. Preparation of T−DBA

**Scheme 1:**
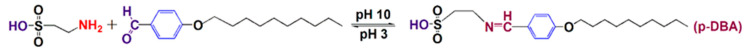
Principle of synthesizing the dynamic covalent surfactant (T−DBA).

Taurine (1.00 g) and DBA (2.09 g) were mixed in CH_3_OH with a 1:1 molar ratio of primary amine to aldehyde. The mixture was stirred at 600 rpm for approximately 30 min at room temperature (25 °C) to obtain the dynamic covalent surfactant T−DBA. T−DBA was prepared by a dynamic imine bond between the primary amine groups in the taurine and aldehyde groups in the DBA through a Schiff base reaction (Scheme 1). After completing the reaction, the solvent (CH_3_OH) was removed by rotary evaporation at reduced pressure, with the residue being the product (T−DBA).

### 2.3. Fourier-Transform Infrared (FTIR) Spectroscopy Measurement

FTIR was used to detect the Schiff base reaction between taurine and p-decyloxybenzaldehyde (DBA). The structures of taurine, DBA, and T−DBA were investigated using an FTIR spectrometer (Bruker Optics, Germany). The taurine sample was homogeneously mixed with potassium bromide (KBr) to yield a transparent pellet for FTIR characterization. The DBA and T−DBA samples were prepared by “sandwiching” the samples between KBr windows. The FTIR spectra with baseline correction were obtained at a 4 cm^−1^ wavenumber resolution with 128 scans for analysis.

### 2.4. ^1^H NMR Measurement

The ^1^H NMR spectrum of T−DBA was measured on a Bruker spectrometer (AVANCE 400, Bruker, Karlsruhe, Germany) at room temperature using CD_3_OD as the solvent.

### 2.5. Surface Tension Measurement

The surface tension of T−DBA aqueous solutions was measured using a platinum ring on an automated surface tensiometer (JYW-200, Chengde Dingsheng Testing Machine Testing Equipment Co. Ltd., Chengde, China). The glass cell was cleaned and repeatedly rinsed with water. The platinum ring was flamed to incinerate any organic contaminants before each measurement. The measurement was conducted at room temperature and repeated at least three times for each solution.

### 2.6. Dynamic Interfacial Tension (IFT) Measurement

The dynamic IFT between liquid paraffin and T−DBA aqueous solution with different pH values was measured using a drop profile analysis tensiometer (Tracker, Lyon, France). Images were captured during forming an aqueous droplet (3 µL, T−DBA aqueous solution) in liquid paraffin. The interfacial tension was calculated using the Laplace equation from the curvature of the droplet obtained by analyzing the captured images.

### 2.7. Nanoemulsion Preparation

To prepare nanoemulsions, 1 mL liquid paraffin was transferred to a 10 mL vial containing 5 mL aqueous solution (pH 10) of T−DBA at different concentrations (concerning the total emulsion weight). The mixtures were homogenized by ultrasonication with a SCIENTZ JTY92-IIN sonicator (Ningbo, China). A 6 mm probe at 200 W using 10 pulse sets of 5 s on followed by 5 s off in an ice bath to avoid overheating to constitute the nanoemulsions. The obtained nanoemulsions at pH 10 were sealed in vials and stored at room temperature. The type of nanoemulsions was examined by conductivity measurements and the drop test.

### 2.8. Dynamic Light Scattering (DLS) Measurement

The droplet size and droplet size distribution of the nanoemulsions were characterized by DLS (Brookhaven BI-200SM instrument, Holtsville, NY, USA) measurement. The DLS measurement was performed at a fixed scattering angle of 90° using a green laser (λ = 532 nm) with variable intensity. The hydrodynamic diameter was calculated from autocorrelation functions, which were analyzed with the CONTIN method.

### 2.9. Zeta Potential Measurement

The zeta potential of the nanoemulsions was analyzed using a ZetaPALS zeta potential analyzer (Brookhaven Instrument, Holtsville, NY, USA). To measure the zeta potential, the nanoemulsions were diluted about 2000 times and then placed in the electrophoretic cuvette. The values of the zeta potential were calculated using the Smoluchowski formula.

### 2.10. pH Adjustment Method

The pH value of the nanoemulsion systems was adjusted by adding HCl or KOH to the system, which was monitored by using a Sartorius basic pH meter PB-10.

## 3. Results and Discussion

### 3.1. Preparation and Characterization of T−DBA

We chose taurine with a primary amine group as the hydrophilic part and *p*-decyloxybenzaldehyde with an aldehyde group as the hydrophobic parts of the dynamic covalent small-molecule surfactant (T−DBA). The octanol-water partition coefficient (log P) is one of the most widely used parameters to evaluate the hydrophobicity of organic compounds, and the more positive log P, the higher the hydrophobicity [29]. The theoretically predicated log P values of taurine, DBA, and T−DBA were −1.72, 5.40, and 4.79, respectively (ChemBioDraw Ultra 14, Cambridge Soft, Cambridge, MA, USA). These predictions suggest that taurine is a highly hydrophilic molecule, DBA is a highly hydrophobic molecule, and T−DBA is a relatively hydrophobic molecule. At high pH, the condensation between the primary amine and the aldehyde groups leads to dynamic imine bond formation that undergoes reversible cleavage at low pH [31]. The dynamic nature of imine bonds was exploited to reversibly switch the interfacial activity of T−DBA between on and off states.

**Figure 1:**
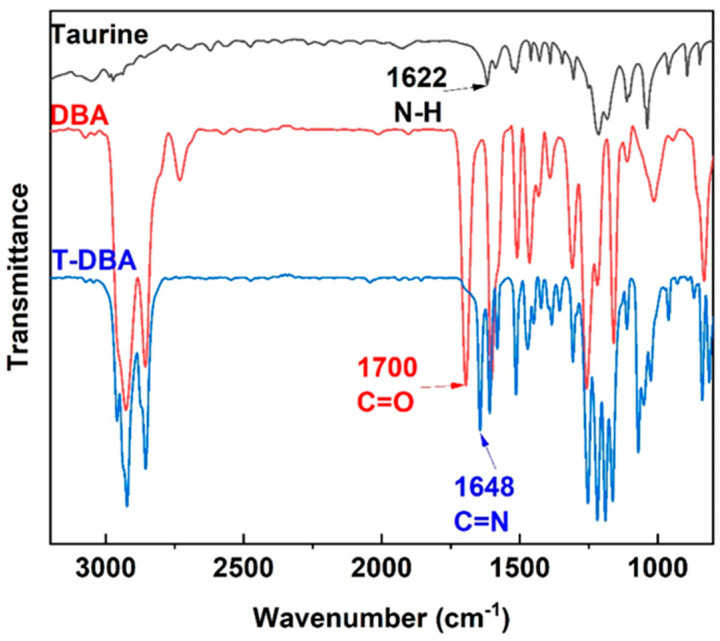
FTIR spectra of taurine (black), DBA (red) and T−DBA (blue).

Information regarding the chemical composition of taurine, DBA, and T−DBA was obtained using FTIR. The FTIR spectrum of taurine in Figure 1 shows a characteristic bending vibration of the primary amine at 1622 cm^−1^. The FTIR spectrum of DBA in Figure 1 shows the characteristic vibration of an aldehyde group (−C=O) at 1700 cm^−1^ [37]. In contrast, the absorption peaks of the bending vibration of the primary amine at 1622 cm^−1^ and the vibration of the aldehyde groups at 1700 cm^−1^ disappear, suggesting that the primary amine group and aldehyde group were reacted. In addition, a pronounced band appeared at 1648 cm^−1^, which is assigned to the stretching vibration of the C=N (imine stretching) bond [38,39,40], indicating that a dynamic imine bond formed between taurine and DBA, and therefore, proving the formation of T−DBA.

**Figure 2:**
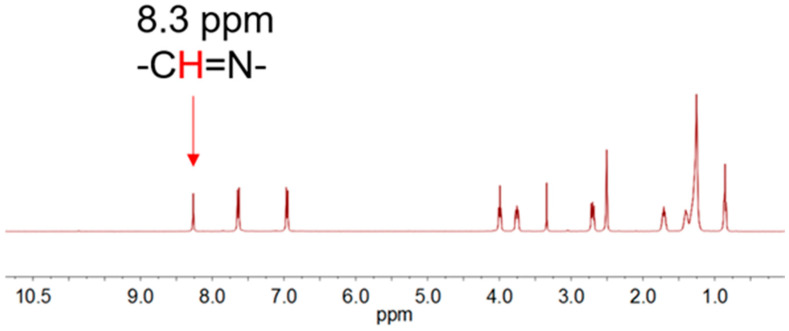
^1^H NMR spectra of T−DBA.

Further evidence of the formation of the T−DBA by dynamic imine bond formation is provided by ^1^H NMR. As shown in Figure 2, the characteristic peak of the dynamic imine bond at 8.3 ppm [38] is clearly visible in the spectrum of T−DBA, demonstrating the formation of T−DBA between taurine and DBA by creating dynamic imine bonds.

### 3.2. The Critical Micelle Concentration (CMC) of T−DBA

**Figure 3:**
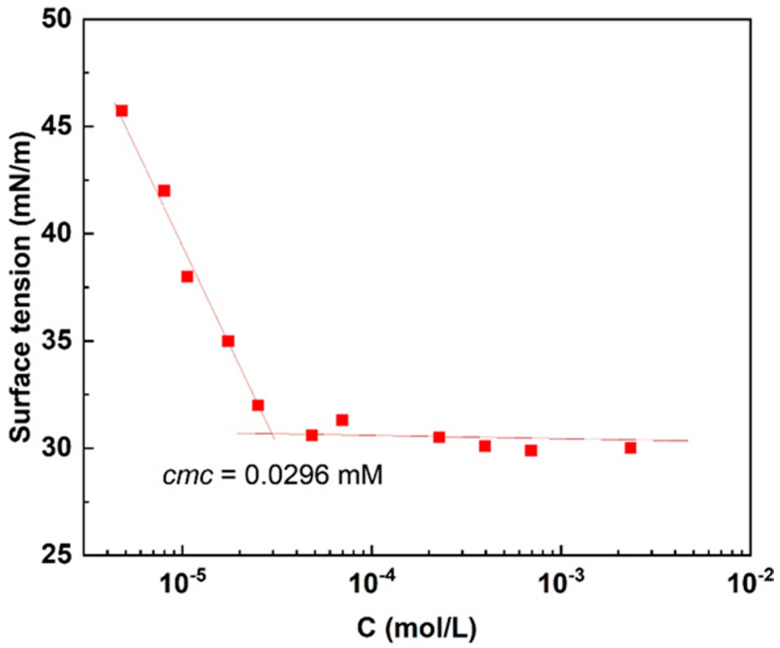
Air-water surface tension of T−DBA at pH 10 as a function of the T−DBA concentration at 298 K.

Taurine is a water-soluble molecule that exhibits little surface activity because of a lack of hydrophobic groups [41]. After modification with DBA, the resulting T−DBA concentrates at the air-water surface reduced the surface tension. The tail of the T−DBA molecule faces the air, and it repels water molecules; the head of the T−DBA molecule remains in the solution, resulting in a reduction of the surface tension at the air-water surface. Increasing the concentration of T−DBA increases the migration of the molecules to the surface, up to a defined concentration (CMC) at which the surface becomes saturated. The T−DBA molecules that remain in the bulk solution at this concentration form micelles through the aggregation of the tails, with the heads of T−DBA forming the outer surface of the micelles. Figure 3 shows a plot of surface tension versus molar concentrations of T−DBA at 298 K. As shown in Figure 3, the CMC value of T−DBA determined from the surface tension–concentration profile is approximately 0.0296 mM, which was smaller than the CMC of conventional surfactants, such as sodium dodecyl sulfate (SDS) (CMC = 8 mM) [42]. At a T−DBA concentration of 0.008 mM (below CMC), the surface tension of T−DBA decreased to 42 mN/m. Above the CMC, the surface tension value reached a value of 30 mN/m. This result indicates that T−DBA molecules have high surface activity.

### 3.3. The Emulsification Ability of the T−DBA Surfactant

**Figure 4:**
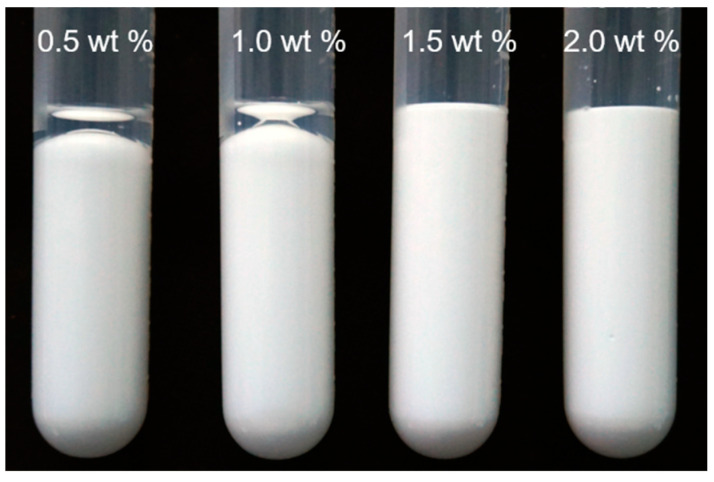
Digital photographs of liquid paraffin in water (1:5, *v/v*); the paraffin nanoemulsions were stabilized by different concentrations (wt %) of T−DBA at pH 10. The photographs were taken 12 h after preparation.

The high surface activity of T−DBA suggests that T−DBA has the potential to stabilize nanoemulsions [6]. To demonstrate the emulsification capabilities of T−DBA, nanoemulsions with different T−DBA concentrations (0.5–2.0 wt %) were prepared (Figure 4). The conductivity measurement and the drop test confirmed that the nanoemulsion was an oil/water (O/W) type. At the T−DBA concentration of 0.5 wt % and 1.0 wt %, the emulsion was not sufficiently stable, and some free oils were observed on the surfaces of the emulsions within 12 h (Figure 4), which was probably due to the insufficient amount of surfactant molecules to fully cover the droplets [43]. With increased T−DBA concentration to 1.5 wt % and 2.0 wt %, stable nanoemulsions were obtained, and no phase separation was observed within 12 h (Figure 4). By increasing the T−DBA concentration, the nanoemulsion stability was increased, which might attribute to the decrease in droplet size [44].

**Figure 5:**
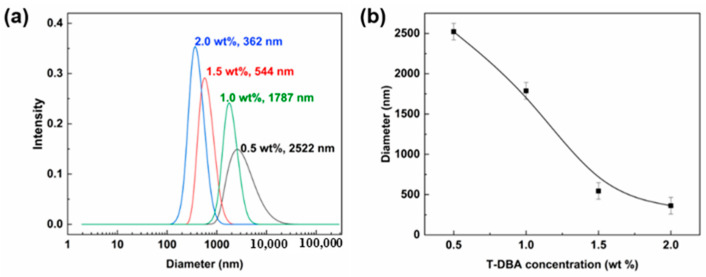
(**a**) Droplet size and droplet size distributions of liquid paraffin in water (1:5, *v/v*); (**b**) Droplet size of nano-emulsions as a function of T−DBA concentration. The paraffin nanoemulsions were prepared at pH 10 with different T−DBA concentrations (wt %). The DLS measurements were carried out 12 h after preparation.

Next, the impact of the T−DBA concentration on the droplet size of liquid paraffin in water nanoemulsions was measured by DLS (Figure 5). With increasing T−DBA concentration from 0.5 wt % to 2.0 wt %, a greater amount of T−DBA covered a larger liquid paraffin-water interface during emulsification, and thus, decreased droplet size occurred. Higher emulsifier concentrations produced smaller droplet sizes [5,44,45]. Therefore, the droplet size of the emulsions decreased from approximately 2522 to 362 nm, accompanied by a narrower droplet size distribution (Figure 5).

It was proposed that the emulsification ability of T−DBA resulted from the surface activity of T−DBA. TEM image of the nanoemulsion reveals the presence of spherical droplets with a mean number-weight diameter of 280 nm (Figure S1). It is worth noting that the diameter determined by DLS is larger than the diameter determined from the TEM image because the diameter determined by DLS includes the hydrated interfacial layer. In the control experiment, we excluded the possibility that taurine itself could not sufficiently stabilize the oil droplets (Figure S2, left) because it is too hydrophilic to strongly attach to the oil droplet surfaces (Figure S3). DBA alone could not produce a stable emulsion either (Figure S2, right) because it is too hydrophobic to strongly attach to the droplet surfaces (Figure S3).

**Figure 6:**
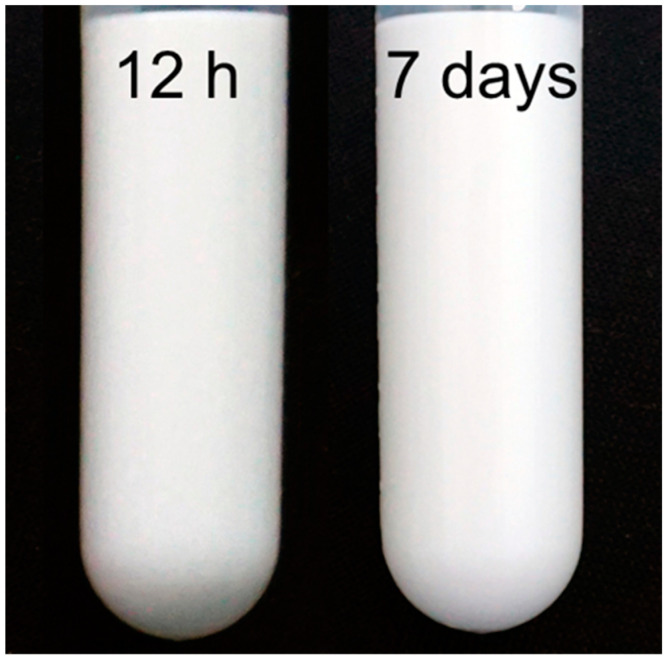
Digital photographs of liquid paraffin in water (1:5, *v/v*); the paraffin nanoemulsions were stabilized by T−DBA (1.5 wt %). The photographs were taken 12 h (**left**) and 7 days (**right**) after preparation.

**Figure 7:**
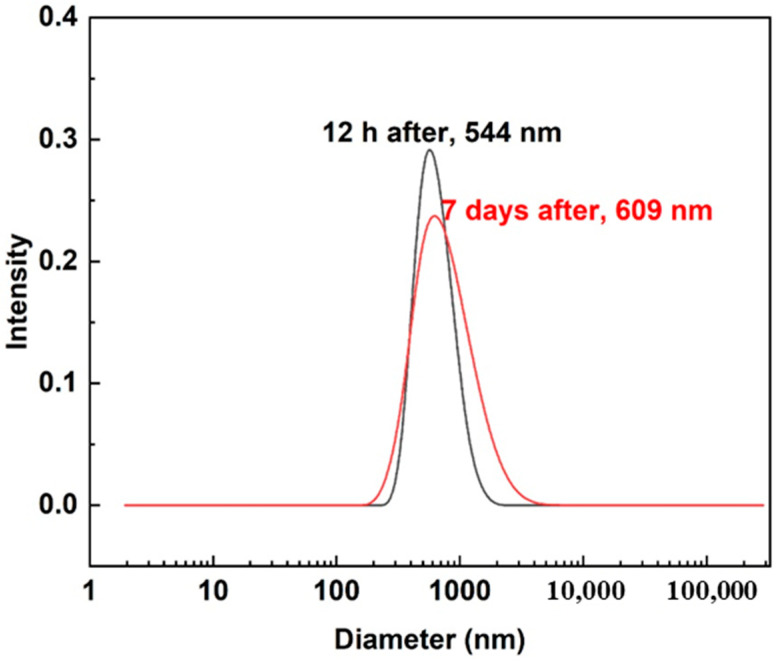
Droplet size and droplet size distribution of liquid paraffin in water (1:5, *v/v*); the nanoemulsion was stabilized by 1.5 wt % T−DBA at pH 10, which were carried out 12 h and 7 days after preparation.

To investigate the long-term stability of the nanoemulsions, 1.5 wt % T−DBA stabilized nanoemulsion was selected as a representation. Satisfyingly, the nanoemulsion at pH 10 showed no noticeable phase separation after 7 days of storage (Figure 6). The nanoemulsion exhibited relatively stable droplet coalescence at pH 10, with little increase in droplet size within 7 days of storage (Figure 7) and no evidence of coalescence. Additionally, the droplet size distribution of the nanoemulsion remained almost unchanged within 7 days of storage (Figure 7). The zeta potential of the nanoemulsion is about −30 mV at pH 10, which suggests that the stability of the nanoemulsion is improved by the electrostatic repulsion mechanism. These results demonstrate that at pH 10, the T−DBA emulsifier effectively stabilized the liquid paraffin in water nanoemulsions.

### 3.4. pH-Responsive Nanoemulsions

**Figure 8:**
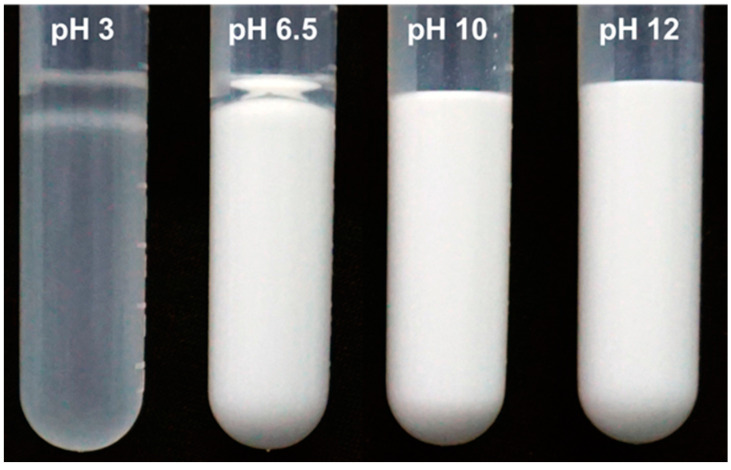
Digital photographs of 1.5 wt % T−DBA-stabilized liquid paraffin in water (1:5, *v/v*); these nanoemulsions were at different pH values (pH 3, 6.5, 10, and 12). The photographs were taken 30 min after changing the system to a particular pH value.

The dynamic nature of the imine bond (formation at high pH and cleavage at low pH) endows the T−DBA with a pH-responsive nature [38,46,47]. Given the pH-representativeness of T−DBA, the obtained nanoemulsions were expected to respond to pH. To illustrate the pH-responsive nature of the nanoemulsions, the effect of pH on the stability of the nanoemulsions was investigated. It was observed that T−DBA-stabilized nanoemulsions became unstable upon lowering the pH. As shown in Figure 8, the T−DBA-stabilized nanoemulsion was very stable at pH 12 and 10. When the pH was decreased to 6.5, the phase separation was visible within 30 min (Figure 8), which indicated dramatic coarsening. After further decreasing the pH to 3.0, droplet breakup and coalescence completely occurred within 30 min (Figure 8), and complete phase separation was achieved. In summary, when the pH was decreased from 12 to 3, the T−DBA stabilized nanoemulsion underwent a transition from the stable to the unstable state.

**Figure 9:**
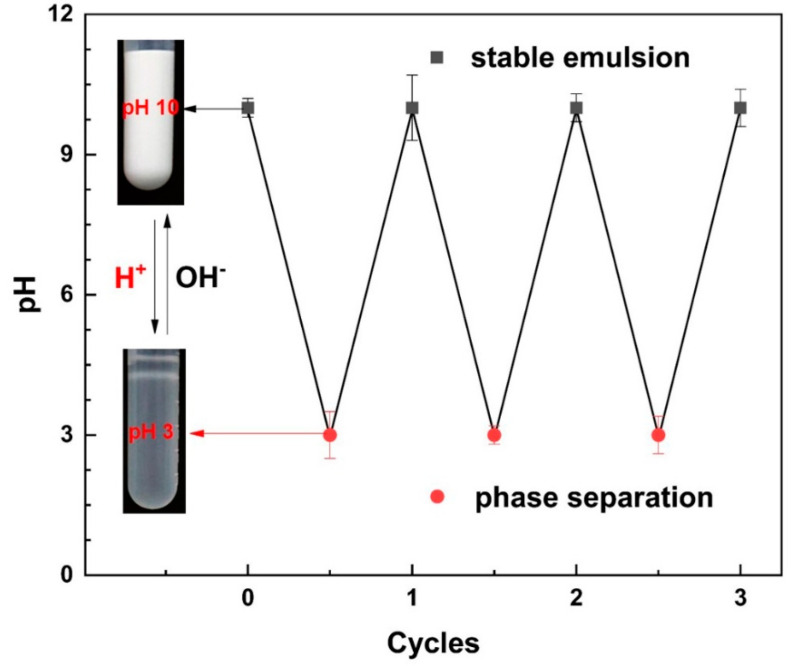
pH-triggered emulsification/demulsification processes, with the inset showing digital photographs of the 1.5 wt % T−DBA-stabilized nanoemulsion (1:5, *v/v*) at pH 10 (**top**) and pH 3 (**bottom**).

At pH 10, a stable nanoemulsion was obtained, as discussed in Section 3.3 (Figure 6 and Figure 7). When the pH was decreased from 10 to 3, the droplet size gradually increased, displaying sensitivity to pH. Initially, the droplet size of the nanoemulsion was approximately 544 nm (Figure 5). Then it increased to approximately 100 μm after 10 min (Figure S4). After extending the treatment to 20 min, no effective data were obtained because there was insufficient droplets. After approximately 30 min, complete demulsification was nearly achieved (inset in Figure 9, top).

It should be noted that a stable and homogenous nanoemulsion cannot be prepared after re-sonication (Figure S5). However, upon increasing the pH from 3 to 10, a stable nanoemulsion was obtained after sonication (inset in Figure 9, bottom). It was evident that the nanoemulsion can be switched between a stable and unstable state via a variation in pH. Additionally, the pH-induced reversible process of emulsification/demulsification can be repeated for at least 3 cycles (Figure 9). More importantly, the type and droplet size of the regenerated nanoemulsion was almost the same as the original nanoemulsion (Figure S6), suggesting that the emulsifying performance of T−DBA remained unchanged throughout all 3 cycles.

### 3.5. Mechanism of pH-Responsive Nanoemulsions

**Figure 10:**
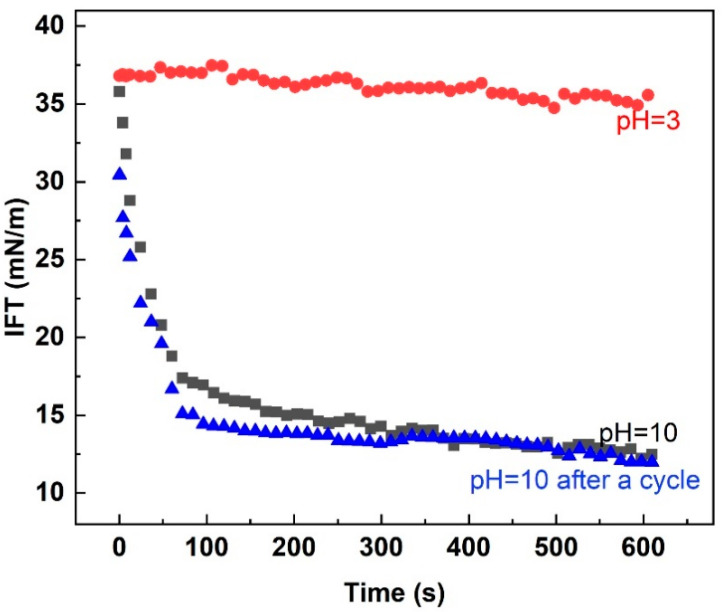
Interfacial tension between liquid paraffin and T−DBA aqueous solution (0.1 mM) at different pH values (blue denotes pH 10, red denotes pH 3, and black denotes changing the pH back to 10).

To show the pH-responsive behavior of the nanoemulsion, the interfacial tension between the liquid paraffin and the T−DBA aqueous solution at different pH values was monitored at room temperature. The results in Figure 10 reveal a low interfacial tension of 10.3 mN·m^−1^ after a 10 min equilibration at pH 10, indicating that T−DBA is in an active interfacial state. Such interfacial activity of T−DBA is attributed to introducing hydrophobic benzene rings and decyl chains through forming dynamic covalent bonds between hydrophilic taurine and hydrophobic DBA.

**Figure 11:**
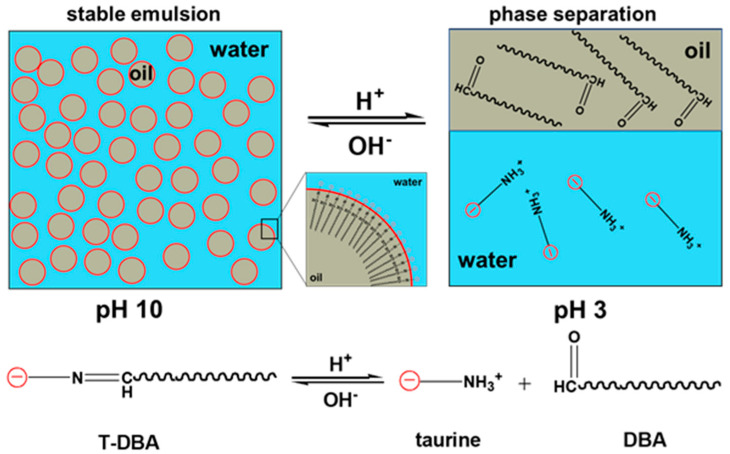
Schematic diagram of the emulsification/demulsification cycles of the pH-responsive liquid paraffin in water nanoemulsions stabilized by T−DBA.

Because of the interfacial activity, the T−DBA adsorbs to the oil–water interface to stabilize the droplets (Figure 11, left). Chen et al. reported that an emulsifier with an interfacial tension of 10 mN/m was sufficient to adsorb on the oil–water interface and stabilize the emulsions [29]. As the pH decreased from 10 to 3, the IFT increased to 34 mN·m^−1^ (Figure 10), which was similar to the interfacial tension between liquid paraffin and pure water (36 mN·m^−1^), DBA in liquid paraffin and pure water (34.8 mN·m^−1^), and liquid paraffin and the taurine aqueous solution (31.2 mN·m^−1^) (Figure S3). These results indicate that the decomposition of interfacial active T−DBA into interfacial inactive taurine and DBA occurred due to the decomposition of the dynamic imine bond [46,47]. Therefore, the interfacial inactive taurine and DBA desorb from the droplet interface, which indicates that no active interfacial agent is remaining at the oil–water interface. Intense coalescence of the droplets and complete phase separation was allowed to occur (Figure 11, right).

Hydrophilic taurine desorbs from the interface to the aqueous phase, and hydrophobic DBA desorbs from the interface to the liquid paraffin phase. Upon increasing the pH to 10, the equilibrium IFT was returned to 10.5 mN·m^−1^ (Figure 10), indicating that reformation of interfacial active T−DBA occurred in situ (Figure 11, left). The reformed interfacial active T−DBA was adsorbed into the oil–water interface again after resonication. A stable nanoemulsion was obtained once more. The essence of the pH responsiveness of the nanoemulsion is the reversibility of the dynamic imine bond in the T−DBA emulsifier.

### 3.6. Universality of This Strategy

**Figure 12:**
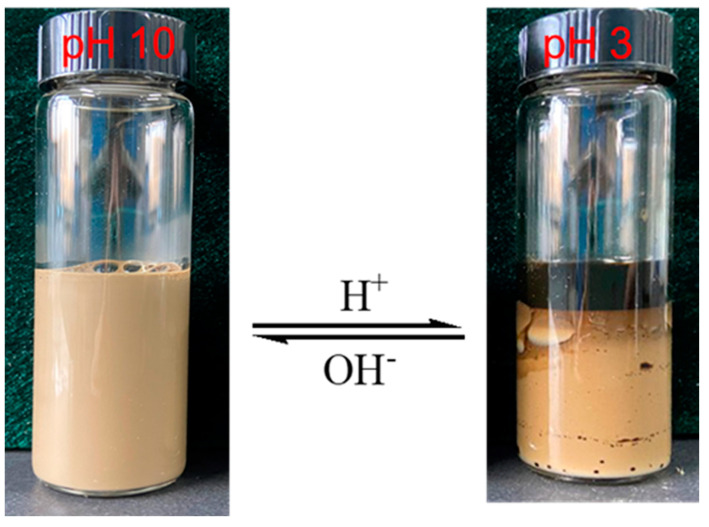
Digital photographs of 1.5 wt % T−DBA stabilized crude oil in water (1:5, *v/v*) nanoemulsion; the nanoemulsions are shown at pH 10 12 h after preparation (**left**) and at pH 3 (**right**) followed by a period of standing for 30 min.

Nanoemulsions help labilize oil by lowering the oil–water interfacial tension in EOR applications. However, a stable emulsion is only useful during one stage of the process, after which the stable emulsion becomes a liability that hinders separation of the components. Therefore, emulsions with controllable stability are needed in EOR applications. To explore the potential EOR applications of the dynamic covalent small-molecule surfactant, crude oil was selected as the oil phase used in a water nanoemulsion. Crude oil in a water nanoemulsion was obtained (Figure 12, left) with droplet sizes of 645 nm (Figure S7). Moreover, the crude oil in the water nanoemulsion was stable after 7 days of storage without any creaming or coalescence.

Although high emulsion stability is desirable for the recovery of the emulsified crude oil, effective demulsification of the crude oil in a water nanoemulsion after subterranean production is also important for the subsequent transportation and refining of crude oil. The demulsification of the crude oil in a water nanoemulsion can be easily achieved by pH variation. By decreasing the pH from 10 to 3, the crude oil in the water nanoemulsion began to coalesce and was completely demulsified within 30 min (Figure 12, right). This performance confirms that pH can be specifically used to switch the crude oil on and off in the water nanoemulsion with T−DBA as the emulsifier. Such a pH-responsive nature is highly desirable in EOR applications.

Three oils (*n*-tetradecane, toluene, and dichloromethane) were chosen for preparing nanoemulsions. The parameters of the oils are listed in Table 1. The polarity can be characterized by the dielectric constant. *n*-Tetradecane and toluene are weakly polar oils, and dichloromethane is a highly polar oil. Liquid paraffin has a high viscosity of approximately 40 cP, but the other oils are near 1 cP. In all of the cases, stable nanoemulsions were obtained, regardless of the polarity and viscosity of the oils. The corresponding nanoemulsions are shown in Figure S8a. T−DBA stabilized a broad range of oils in water nanoemulsions, which is important for further potential applications. All three types of nanoemulsions were switched on and off by regulating the pH values (Figure S8a,b). Moreover, the type and droplet size of the regenerated nanoemulsions remained almost unchanged compared to the original one.

To demonstrate the generality of our result, additional dynamic covalent surfactants were prepared with taurine and several aromatic aldehydes: p-octyloxybenzaldehyde (p-OBA), p-tetradecyloxybenzaldehyde (p-TBA), or p-hexadecyloxybenzaldehyde (p-HBA). Using these dynamic covalent small-molecule surfactants, we could successfully obtain pH-responsive nanoemulsions (Figure S9). Therefore, this method provides a general technique for successfully preparing pH-responsive nanoemulsions based on dynamic covalent small-molecule surfactants.

## 4. Conclusions

Dynamic covalent small-molecule surfactants (T−DBA) were prepared using the small molecules taurine and p-decyloxybenzaldehyde (DBA). The primary amine group in the taurine and the aldehyde group in the DBA were reacted by forming a dynamic imine bond. FTIR and ^1^H NMR proved the successful formation of T−DBA. Though similar methods have been used to prepare emulsifiers, pH-responsive macroemulsions were obtained using these emulsifiers as stabilizers. The emulsifier obtained in this work can be used to prepare pH-responsive nanoemulsions. At pH 10, T−DBA effectively reduced the interfacial tension between oil and water, which was used to prepare stable nanoemulsions. However, upon decreasing the pH from 10 to 3, T−DBA decomposed, which induced the loss of interfacial activity and led to the demulsification of nanoemulsions. The responsiveness of the nanoemulsion originates from the pH responsiveness of the dynamic covalent bond in the T−DBA. The crude oil in the water nanoemulsion based on a dynamic covalent small-molecule surfactant was demulsified by decreasing the pH from 10 to 3. Such pH-responsive nanoemulsions have potential utility in EOR applications.

## Figures and Tables

**Table 1:** Dielectric constant and viscosity of the oils.

Oil	Liquid Paraffin	*n*-Tetradecane	Toluene	Dichloromethane
Dielectric constant	1.9	2.03	2.4	9.1
Viscosity (cP, 20 °C)	40	1.37	0.59	0.44

## Supplementary Materials

The following are available online at https://www.mdpi.com/2079-4991/11/6/1390/s1. Figure S1: Transmission Electron Microscope (TEM) image of nano-emulsion stabilized by 2.0 wt % taurine–p-decyloxybenzaldehyde (T−DBA) at pH 10. Figure S2: Optical photographs of emulsions (immediately after sonication) stabilized by 1.5 wt % taurine (left) and 1.5 wt % pDBA (right) with volume ratio of liquid paraffin to water of 1:5. Figure S3: Dynamic interfacial tension (IFT) of liquid paraffin-pure water, DBA liquid paraffin solution-pure water, and liquid paraffin-taurine aqueous solution. The concentration of DBA in the liquid paraffin phase is 0.1 mM and taurine in the aqueous phase is 0.1 mM. Figure S4: Optical micrograph of the liquid paraffin in water emulsion taken 10 min after changing the pH from 10 to 3. Figure S5: Photograph of the phase-separated system after re-sonication at pH 3. Figure S6: Droplet size and droplet size distribution curves for the nanoemulsions prepared with 1.5 wt % T−DBA at pH 10, initially prepared and after 3 emulsification/demulsification cycles. Figure S7: Droplet size and droplet size distribution curve for the crude oil in water nanoemulsions prepared with 1.5 wt % T−DBA at pH 10. Figure S8: Photographs of 1.5 wt % T−DBA stabilized oils in water (1:5, *v/v*) nanoemulsions with different types of oil at pH 10 (a) and at pH 3 (b). Figure S9: Photographs of 1.5 wt % T−OBA-, T−TBA-, T−HBA-, or T−HBA-stabilized liquid paraffin in water (1:5, *v/v*) nanoemulsions atpH 10 (a) and pH 3 (b) (PDF).
