# Supplementary material for: pH-Responsive Nanoemulsions Based on a Dynamic Covalent Surfactant
Source: Nanomaterials (Basel). 2021 May 25;11(6):1390. doi: 10.3390/nano11061390 (PMC8227844; doi:10.3390/nano11061390)
Supplement: Supplementary file 1 [file nanomaterials-11-01390-s001.zip › nanomaterials-1229736-supplementary.pdf]

nanomaterials

pH-Responsive Nanoemulsions Based on a Dynamic Covalent Surfactant

Gaihuan Ren, Bo Li, Lulu Ren, Dongxu Lu, Pan Zhang, Lulu Tian, Wenwen Di, Weili Shao,* Jianxin He,* and Dejun Sun*

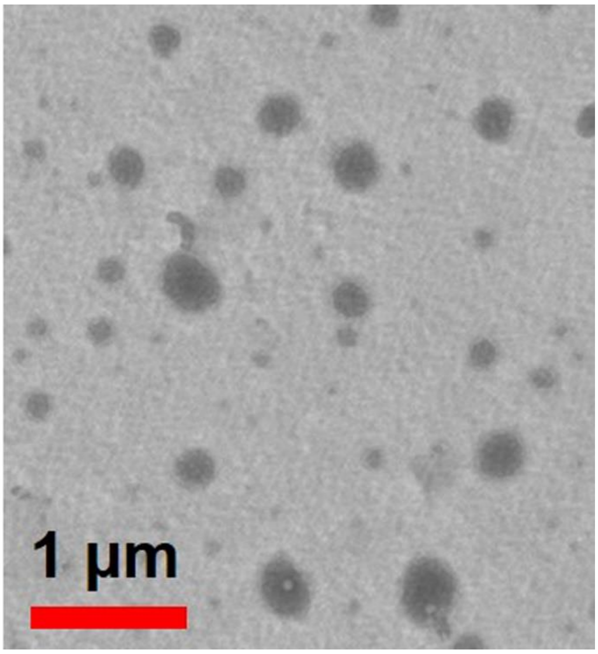

Figure S1. Transmission Electron Microscope (TEM) image of nano-emulsion stabilized by 2.0 wt % taurine–p-decyloxybenzaldehyde (T−DBA) at pH 10.

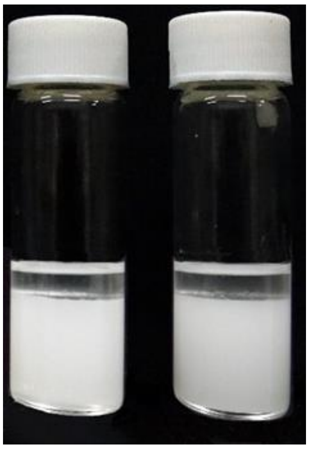

Figure S2. Optical photographs of emulsions (immediately after sonication) stabilized by 1.5 wt % taurine (left) and 1.5 wt % pDBA (right) with volume ratio of liquid paraffin to water of 1:5.

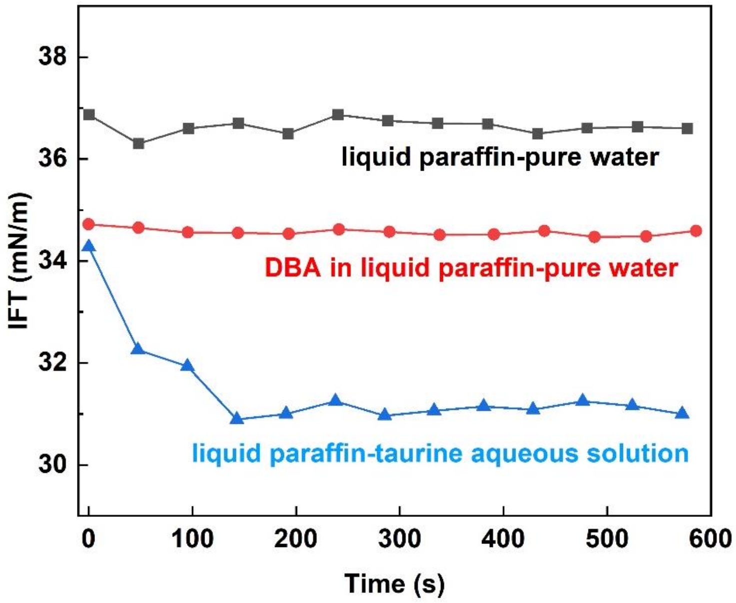

Figure S3. Dynamic interfacial tension (IFT) of liquid paraffin-pure water, DBA liquid paraffin solution-pure water, and liquid paraffin-taurine aqueous solution. The concentration of DBA in the liquid paraffin phase is 0.1 mM and taurine in the aqueous phase is 0.1 mM.

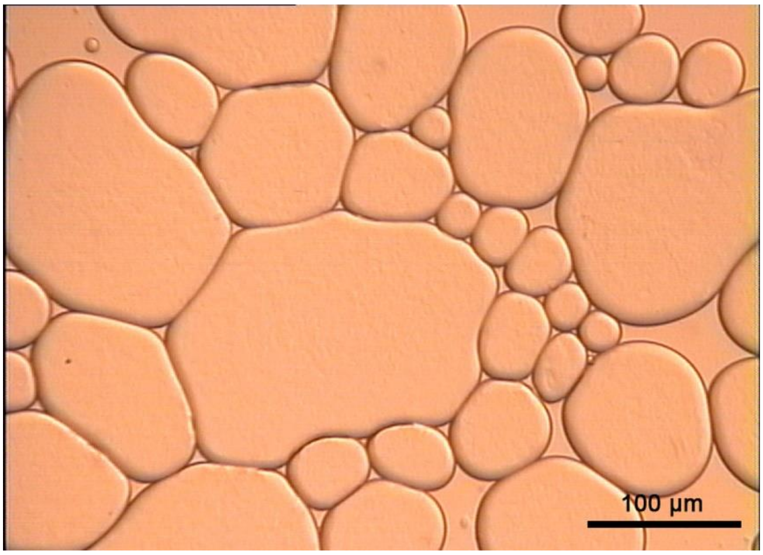

Figure S4 Optical micrograph of the liquid paraffin in water (1:5, *v*/*v*) emulsion taken 10 min after changing the pH from 10 to 3. The emulsion was prepared 1.5 wt % T−DBA at pH 10.

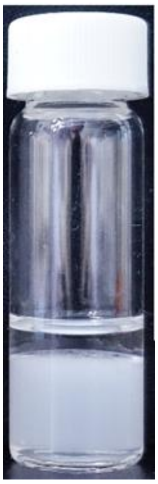

Figure S5. Photograph of phase separated system after re-sonication at pH 3.

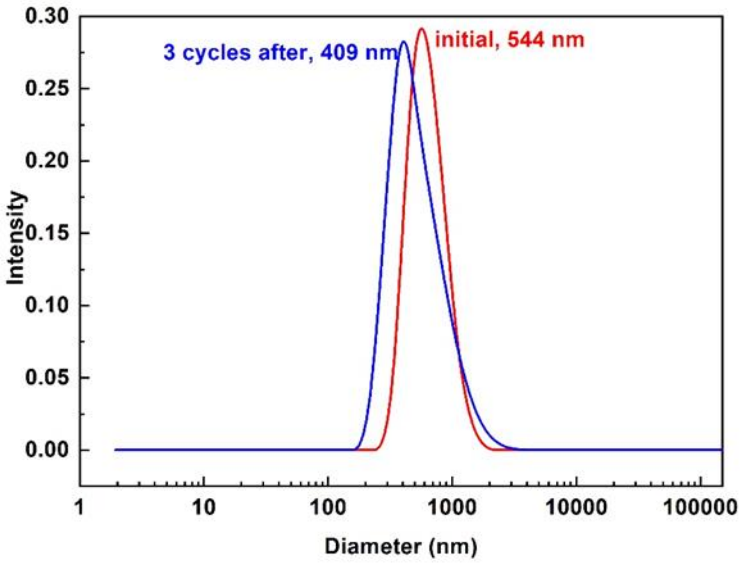

Figure S6. Droplet size and droplet size distribution curves for the liquid paraffin in water (1:5, *v*/*v*) nano-emulsions prepared with 1.5 wt % T−DBA at pH 10, initially prepared and after 3 emulsification/demulsification cycles.

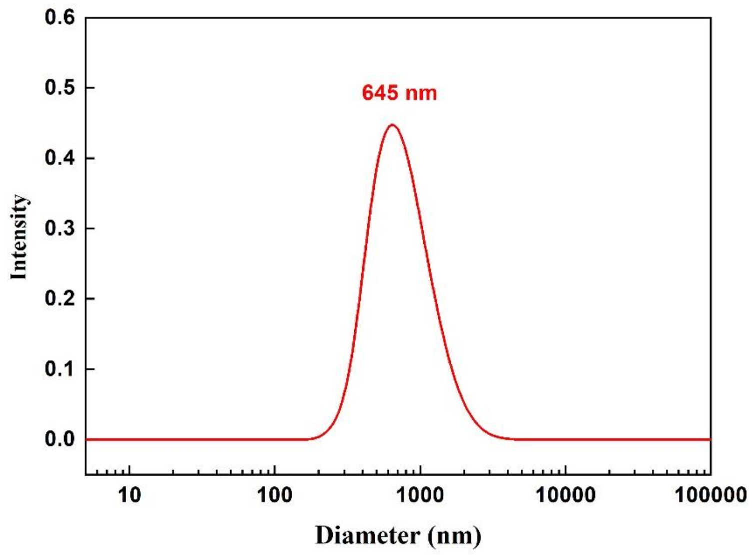

Figure S7. Droplet size and droplet size distribution curve for the crude oil in water nano-emulsions prepared with 1.5 wt % T–DBA at pH 10.

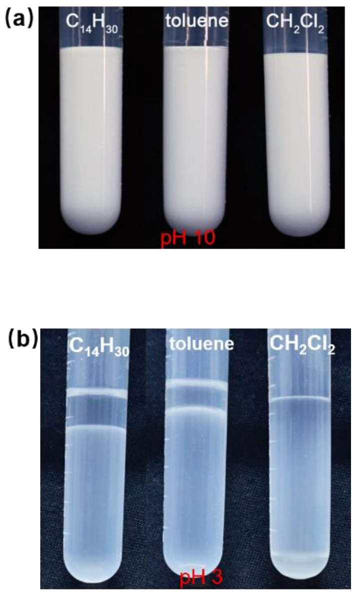

Figure S8. Photographs of 1.5 wt % T–DBA stabilized oil in water (1:5, *v*/*v*) nano-emulsions with different types of oil at pH 10 (a, stable emulsion) and at pH 3 (b, 30 min after adding HCl, complete phase separation). Photograph (**a**) was taken 12 h after the initial prepared nano-emulsion, photograph (**b**) was taken 30 min after the decreasing pH from 10 to 3.

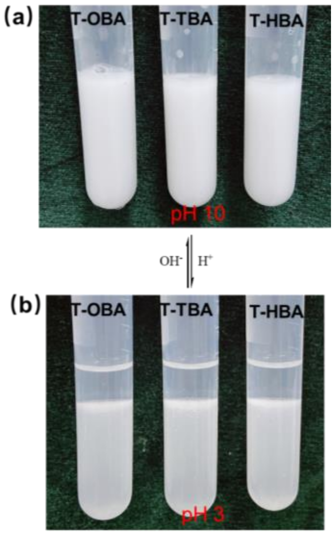

Figure S9. Photographs of 1.5 wt% T–OBA, T–TBA or T–HBA stabilized liquid paraffin in water (1:5, *v*/*v*) nano-emulsions at pH 10 (a, stable nano-emulsion) and at pH 3 (b, 30 min after adding HCl, complete phase separation). Photograph (**a**) was taken 12 h after the initial prepared emulsion, photograph (**b**) was taken 30 min after the decreasing the pH from 10 to 3.
